# Assessment of genetic diversity among Iranian *Aegilops triuncialis* accessions using ISSR, SCoT, and CBDP markers

**DOI:** 10.1186/s43141-020-00107-w

**Published:** 2021-01-11

**Authors:** Lavin Khodaee, Reza Azizinezhad, Ali Reza Etminan, Mahmoud Khosroshahi

**Affiliations:** 1 Department of Biotechnology and Plant Breeding, Islamic Azad University Science and Research Branch, Tehran, Iran; 2 Department of Plant Breeding and Biotechnology, Kermanshah Branch, Islamic Azad University, Kermanshah, Iran

**Keywords:** *Ae. Triuncialis*, Genetic diversity, Polymorphism, Wild wheat, Iran

## Abstract

**Background:**

Crop wild relatives (CWRs) are commonly used as a suitable genetic reservoir for plant breeding. They can be used for enhancing the tolerance of plant varieties to biotic and abiotic stresses. Studying the genetic diversity of related wheat species in Iran could be useful to improve different traits of bread wheat, since the country is one of the major centers of genetic diversity and distribution of Aegilops species. Therefore, the aim of the present study was to determine the relationship among 48 *Aegilops triuncialis* accessions using three DNA marker systems, including start codon targeted (SCoT), CAAT box-derived polymorphism (CBDP), and inter-simple sequence repeat (ISSR) markers.

**Results:**

A total of 359 amplified DNA fragments were generated using 13 CBDP, 14 SCoT, and 16 ISSR primers that produced 96, 147, and 152 bands, respectively. The discriminating power of the three markers was assessed using polymorphism information content (PIC), marker index (MI), and resolving power (Rp). The mean values of PIC for ISSR, SCoT, and CBDP markers were 0.3, 0.26, and 0.34, respectively, indicating the efficiency of the three markers in detecting polymorphism among the studied accessions. ISSR markers had the highest values of MI, Rp, and polymorphism percentage as compared to SCoT and CBDP markers. Based on the Shannon index and heterozygosity values, genetic diversity in the Alborz population was more than in other populations. The accessions were classified into six, five, and five groups based on ISSR, SCoT, and CBDP using the UPGMA method. According to the results of cluster and PCoA analyses, the variation patterns corresponded with the geographical distribution of the *Ae. triuncialis* accessions.

**Conclusions:**

The three markers provided a comprehensive pattern of the genetic diversity among the Iranian *Ae. triuncialis* accessions. This information could allow for a future insight into wheat breeding programs.

## Background

Crop wild relatives (CWRs) are a useful reservoir of beneficial genes and alleles that can be used for improving and adjusting varieties that are better suited and resistant to biotic and abiotic stress [1, 2]. Due to global limitations on further land and water resources, plant breeders try to develop new plant varieties using CWRs. However, many CWRs are not adequately collected and are not currently conserved in gene banks across the world [3]. Wild relatives of common wheat (*Triticum aestivum* L.) are progenitors of precious genetic materials that can assist in breeding programs of wheat [4]. Interspecific crosses between wheat and its close wild relatives such as *Aegilops* species are useful for introducing desirable traits that can contribute to improvements in the germplasm of bread wheat [5, 6]. Iran is one of the major centers of genetic diversity and distribution of related wheat species [7]. While many biotic and abiotic stresses affect the quality and quantity of crops in Iran [8, 9], drought stress is one of the major environmental constraints that threaten plant survival, growth, and final yield. The *Aegilops* genus from the Poaceae family consists of 22 species which are native to Eurasia and North Africa. In fact, *Ae. triuncialis* is an allotetraploid with a UUCC genomic formula (2*n* = 28) and with a basic chromosome number (*x* = *n* = 7), whereas bread wheat is an allohexaploid. The U genome played a major role in the evolution of domestic wheat [10].

The plant is resistant to various stresses such as drought and high salinity. It can be a suitable candidate to enable crosses with bread wheat so as to obtain new lines with high levels of drought stress tolerance [11]. Therefore, studying genetic diversity among the accessions of *Ae. triuncialis* could be useful for improving different traits in bread wheat.

Molecular techniques have added a new dimension to scientific research and have offered powerful pathways to identify and manipulate various genes [12]. Recently, molecular markers have played a major role in estimating diversity and evolutionary relationships. They can exclusively be effective for quantifying genetic diversity within plant species. They can be used for identifying and recognizing closely related genotypes [13]. In the past few years, many new gene-related target marker techniques have emerged. Two of these techniques are CBDP and SCoT [14]. SCoT markers are a new type of DNA markers that use single 18-mer primers based on short conserved regions that are close to the start codon ATG [15].

CBDP markers enable the CAAT box region of the plant gene promoters by using a single primer in PCR, similar to RAPD [16]. These two new markers display a higher reproducibility and can generate more information related to biological traits compared with random DNA markers [16]. Furthermore, the markers are novel, accessible, cost effective, and, generally, there is no requirement of prior sequence information of the genome. These markers represent a high level of polymorphism and efficiency. They are successfully utilized in different plant species such as *Cicer* species [17], bread wheat [18], date palm [18], and walnut [19]. ISSR markers are one of the most efficient marker systems because of their capacity to reveal several informative bands from a single amplification [20]. The marker has a high polymorphic degree among closely related varieties, along with high levels of repeatability and reliability which are efficiently used for assessing genetic variation in different crops such as *Achillea* species [21, 22], melon [23], *Zingiber officinale* [24], and cowpea [25]. Shortages in water resources are expected sooner or later in many parts of the world due to climate change and overpopulation [26].

Crosses between wheat and its close wild relatives such as *Ae. triuncialis* can be useful to obtain new drought-resistant varieties. Therefore, the present study was performed to evaluate the genetic diversity and relationships among *Ae. triuncialis* genotypes collected from different regions of Iran. The evaluations of genetic diversity and genetic relationships were based on SCoT, CBDP, and ISSR markers.

## Methods

### Plant materials and DNA extraction

Forty-eight accessions of *Ae. triuncialis* were collected from different geographical regions of Iran. Information on coordinate and habitat characteristics of the sampling locations are presented in Table 1.

**Table 1:** Information of 48 Iranian *Ae. triuncialis* accessions investigated in the present study

Genotype	Origin	Altitude	Longitude	Latitude
G1-G13	Karaj, Alborz, Iran	1312	35° 82′ E	50° 97′ N
G14	Ilam, Ilam, Iran	1369	33° 63′ E	46° 40′ N
G15-G17	Karaj, Alborz, Iran	1312	35° 82′ E	50° 97′ N
G18-G27	Ghazvin, Ghazvin, Iran	1310	36° 27′ E	50° 00′ N
G28	Ilam, Ilam, Iran	1369	33° 63′ E	46° 40′ N
G29	Ghazvin, Ghazvin, Iran	1310	36° 27′ E	50° 00′ N
G30	Karaj, Alborz, Iran	1312	35° 82′ E	50° 97′ N
G31	Ghazvin, Ghazvin, Iran	1310	36° 27′ E	50° 00′ N
G32	Jadeh Darya, Iran			
G33	Mahabad, West Azerbaijan, Iran	1316	36° 76′ E	45° 72′ N
G34	Banab, East Azerbaijan, Iran	1290	37° 33′ E	46° 05′ N
G35	Malekan, East Azerbaijan, Iran	1300	37° 15′E	46° 10′ N
G36	Banab, East Azerbaijan, Iran	1290	37° 33′ E	46° 05′ N
G37	Mahabad, West Azerbaijan, Iran	1316	36° 76′ E	45° 72′ N
G38	Malekan, East Azerbaijan, Iran	1300	37° 15′ E	46° 10′ N
G39-G40	Bokan, West Azerbaijan, Iran	1340	36° 51′ E	46° 20′ N
G41	Urmia, West Azarbaijan, Iran	1348	37° 55′ E	45° 07′ N
G42-G43	Miandoab, West Azerbaijan, Iran	1295	36° 96′ E	46° 10′ N
G44	Tabriz, East Azarbaijan, Iran	1402	38° 07′ E	46° 30′ N
G45	Babol, Mazandaran, Iran	4	36° 53′ E	52° 67′ N
G46	Tabriz, East Azarbaijan, Iran	1402	38° 07′ E	46° 30′ N
G47	Urmia, West Azarbaijan, Iran	1348	37° 55′ E	45° 07′ N
G48	Unknown	-	-	-

The genomic DNAs were isolated from the young leaves of 2-week-old seedlings and were sampled from all genotypes based on the CTAB method [27]. The quality and quantity of the extracted DNAs were determined by both spectrophotometry and agarose gel electrophoresis.

### ISSR-PCR analysis

A set of 16 ISSR primers were used for amplifying the genomic DNA of the accessions (Table 2). The PCR reactions were performed in a 20 μl volume containing 10 μl master mix 2X PCR (ready-to-use PCR master mix 2X; Ampliqon), 6 μl double distilled water, 2 μl template DNA from each samples, and 1 μl of each forward and reverse primers (10 pmol/μl). The amplifications were carried out at 94 °C for 4 min, followed by 35 cycles of denaturation at 94 °C for 30 s, primer annealing at 49.2–54.8 °C for 45 s and primer elongation at 72 °C for 2 min, with a final extension at 72 °C for 7 min using a Bio-Rad (T100) thermal cycler. The DNA was diluted to a concentration of 50 ng/μl to be used in the assay.

**Table 2:** N, NP, Rp, PIC, and MI indexes in *Ae. triuncialis* genotypes produced by ISSR primers

Primer		Primer sequence	N	NP	Rp	PIC	MI
1	IS1	DBDACACACACACACACA	6	6	1.79	0.25	1.50
2	IS3	GACAGACAGACAGACA	11	11	5.75	0.34	3.74
3	IS4	AGAGAGAGAGAGAGAGYT	14	14	4.33	0.23	3.22
4	IS5	ACACACACACACACACC	7	7	4.38	0.39	2.73
5	IS6	GAGAGAGAGAGAGAGARC	13	12	4.00	0.24	2.65
6	IS7	CTCTCTCTCTCTCTCTG	12	12	6.25	0.36	4.32
7	IS9	CACACACACACACACAG	12	11	3.04	0.23	2.31
8	IS11	ACACACACACACACACYA	10	10	2.71	0.22	2.2
9	IS12	GTGTGTGTGTGTGTGTYG	7	7	2.71	0.29	2.03
10	IS13	GAGAGAGAGAGAGAGAYC	7	6	2.67	0.30	1.54
11	IS14	AGAGAGAGAGAGAGAGT	10	9	2.17	0.20	1.62
12	IS15	ACACACACACACACACYG	8	7	2.67	0.25	1.53
13	IS23	CTCTCTCTCTCTCTCTRC	11	9	5.00	0.37	2.72
14	IS25	CACACACACACACACARG	13	11	4.58	0.29	2.67
15	IS27	TGTGTGTGTGTGTGTGRC	10	9	6.21	0.41	3.32
16	IS28	TCTCTCTCTCTCTCTCG	11	11	7.96	0.42	4.62
		Mean		9.5	4.14	0.30	2.67

*N* total number, *NP* number of polymorphic bands, *Rp* resolving power, *PIC* polymorphic information content, *MI* marker index

### SCoT-PCR and CBDP-PCR analyses

Fourteen SCoT primers and thirteen CBDP primers were selected to study genetic diversity among the accessions. The PCR reactions for SCoT markers were programmed by adhering to the following procedure: after initial denaturation of DNA at 94 °C for 5 min, 35 cycles of DNA denaturation at 94 °C occurred for 30 s, primer annealing occurred at 53.7–60.5 °C for 45 s and at 72 °C for 2 min, before a final extension at 72 °C for 7 min. The PCR condition for CBDP markers was set as follows: 5 min of an initial denaturing at 94 °C, 30 cycles of denaturing at 94 °C for 30 s, primer annealing at 49.1–56 °C for 45 s, and primer elongation at 72 °C for 2 min, with a final extension stage at 72 °C for 10 min.

### Visualization of amplified fragments and data analysis

The PCR products were laid out on 1.5% agarose gels using electrophoresis. They were post-stained with SafeView-IITM. Bands were visualized under UV light by gel documentation.

PCR products were visually scored for their presence (1) or absence (0) based on their gel patterns. Statistical analysis for generated data matrices was done by the DARwin computer software [28]. The preferential power of the primers was evaluated using three useful parameters, i.e., polymorphism information content (PIC), Rp, and MI. Accordingly, PIC was calculated according to the formula: 1 − *Sp*_i_^2^ as PIC = 1 − *Sp*_i_^2^ where *p*_i_ is the frequency of the *i*th allele of the locus [29]. MI was calculated according to a formula described by Kumar et al. [30]. The distance coefficient matrix for the three marker data was calculated using the Jaccard distance index. To show the genetic relationships among the studied accessions, a dendrogram was constructed based on the unweighted pair group method with an arithmetic mean algorithm (UPGMA) using the DARwin5.0 software and NTYSYS 2.02 [31]. Using the GenAlEX 6.5 software, a principal coordinate analysis (PCoA) was performed in order to assess the distribution of the accessions. In addition, different genetic diversity indices such as the percentage of polymorphic loci (PPL), the observed (Na) and effective number of alleles (Ne), Nei’s gene diversity (H), and Shannon’s information index (I) were measured by the POPGENE software, version 1.31 [32].

## Results

### Molecular analysis of the markers

**Fig. 1:**
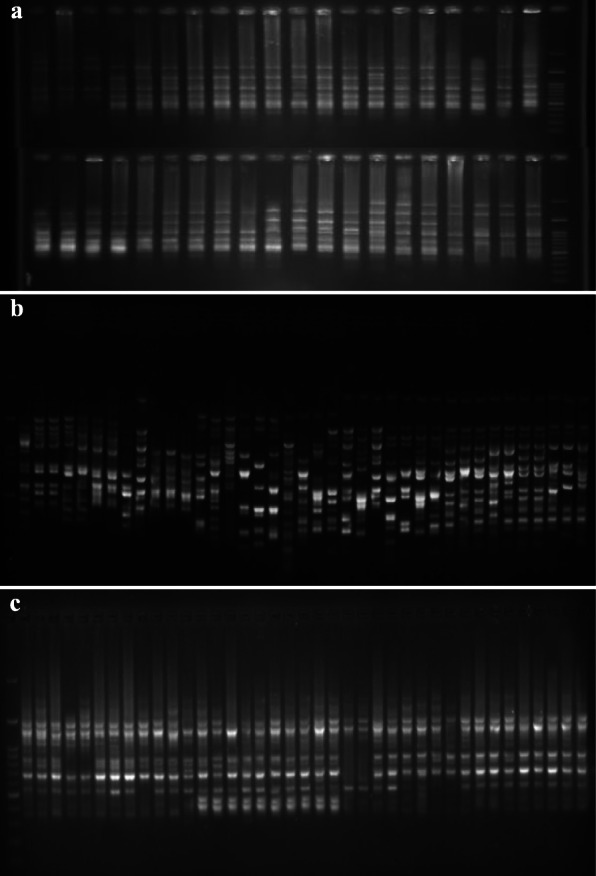
Electrophoretic profile of PCR products using IS13 (**a**), SC9 (**b**), and P10 (**c**) primers for 40 *Ae. triuncialis* genotypes

One representative profile of each marker is shown in Fig. 1. All 16 ISSR primers produced reproducible polymorphic bands, with 152 amplified polymorphic bands generated across the accessions (Table 2). The number of polymorphic bands varied from 6 (IS-8 and IS-13 primers) to 14 bands (IS-4 primer), with an average of 9.5 bands per primer pair and 93.98% polymorphism rate. Also, the PIC ranged from 0.2 (IS-14 primer) to 0.42 (IS-28 primer) with an average of 0.3 per primer pair. The lowest and highest values of MI were observed in IS-1 (1.5) and IS-18 (4.62) primers, respectively. The Rp of the primers varied from 1.79 (IS-1 primer) to 7.96 (IS-28 primer), with a mean value of 4.14.

The SCoT primers generated 162 amplified fragments. Most of them were polymorphic (90.74%). The number of amplified fragments varied from 7 (SC-5, 7, 14) to 14 (SC-2, 9) with an average of 10.5 fragments per primer pair. Furthermore, the average values of PIC, MI, and RP were 0.26, 2.52, and 4, respectively (Table 3). The SC-14 and SC-3 primers revealed the maximum (0.42) and minimum (0.14) of PIC, respectively. In addition, the lowest and the highest MI values were 1.16 and 4.42, being observed in SC-1 and SC-24 primers, respectively.

**Table 3:** N, NP, Rp, PIC, and MI indexes in *Ae. triuncialis* genotypes produced by SCoT primer

Primer		Primer sequence	N	NP	Rp	PIC	MI
1	SC2	CAACAATGGCTACCACCC	14	14	5.83	0.25	3.50
2	SC3	CAACAATGGCTACCACCG	12	11	1.83	0.14	1.41
3	SC4	CAACAATGGCTACCACCT	12	12	5.12	0.29	3.48
4	SC5	CAACAATGGCTACCACGA	7	7	2.62	0.26	1.82
5	SC7	CAACAATGGCTACCACGG	9	7	2.83	0.26	1.41
6	SC8	CAACAATGGCTACCACGT	15	12	3.04	0.18	1.72
7	SC9	CAACAATGGCTACCAGCA	14	14	6.00	0.29	4.06
8	SC11	AAGCAATGGCTACCACCA	11	8	2.25	0.20	1.16
9	SC12	ACGACATGGCGACCAACG	11	11	5.41	0.32	3.52
10	SC14	ACGACATGGCGACCACCG	8	7	4.33	0.42	2.57
11	SC15	ACGACATGGCGACCGCGA	12	12	4.08	0.24	2.88
12	SC21	CACCATGGCTACCACCAT	12	9	2.62	0.23	1.55
13	SC24	CCATGGCTACCACCGCCA	13	13	6.93	0.34	4.42
14	SC26	ACAATGGCTACCACCATC	12	10	3.12	0.22	1.83
		Mean		10.5	4.00	0.26	2.52

*N* total number, *NP* number of polymorphic bands, *Rp* resolving power, *PIC* polymorphic information content, *MI* marker index

The CBDP primers generated 101 bands, 95.05% of which were polymorphic. The number of amplified fragments ranged from 5 (P-5, 7, 8, 15) to 14 (P-14) with an average of 7.38 fragments per primer pair (Table 4). Moreover, the average values of PIC, MI, and Rp for CBDP primers were 0.34, 2.32, and 3.57, respectively. Among different CBDP primers, P-14 primers showed maximum MI (5.04) and Rp (7.21) indices, whereas the minimum values of PIC (0.21), MI (1.28), and Rp (1.88) indices were observed in the P-10 primer.

**Table 4:** N, NP, Rp, PIC, and MI indexes in *Ae. triuncialis* genotypes produced by CBDP primer

Primer		Primer sequence	N	NP	Rp	PIC	MI
1	P1	TGAGCACGATCCAATAGC	8	8	5.17	0.42	3.36
2	P2	TGAGCACGATCCAATAAT	10	9	3.50	0.26	2.10
3	P3	TGAGCACGATCCAATACC	8	7	3.33	0.35	2.14
4	P4	TGAGCACGATCCAATAAG	6	6	3.88	0.39	2.34
5	P5	TGAGCACGATCCAATCTA	5	5	2.42	0.33	1.65
6	P7	TGAGCACGATCCAATCGA	6	5	2.88	0.39	1.62
7	P8	TGAGCACGATCCAATCGG	5	5	3.21	0.41	2.05
8	P9	TGAGCACGATCCAATGAT	7	7	3.13	0.32	2.24
9	P10	TGAGCACGATCCAATGTT	8	7	1.88	0.21	1.28
10	P12	TGAGCACGATCCAATATA	9	9	3.33	0.25	2.25
11	P13	TGAGCACGATCCAATGAG	9	9	2.63	0.25	2.25
12	P14	TGAGCACGATCCAATGCG	14	14	7.21	0.36	5.04
13	P15	TGAGCACGATCCAATTGA	6	5	3.75	0.46	1.91
		MEAN		7.38	3.57	0.34	2.32

*N* total number, *NP* number of polymorphic bands, *Rp* resolving power, *PIC* polymorphic information content, *MI* marker index

### Cluster analysis

**Fig. 2:**
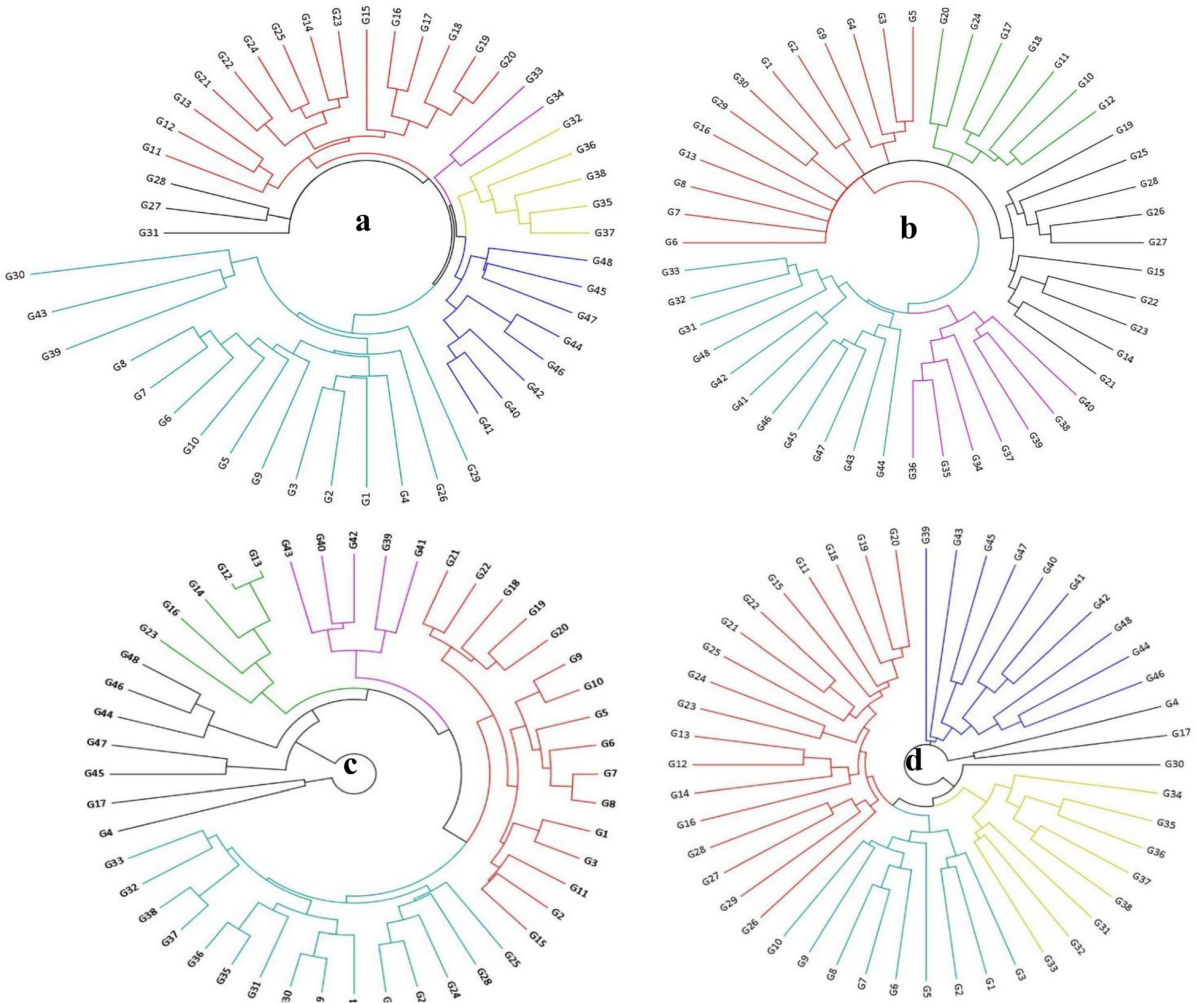
Dendrogram of 48 *Ae. triuncialis* accessions using UPGMA method based on ISSR (**a**), SCoT (**b**), CBDP (**c**), and all the three markers (**d**) data

Cluster analysis involved using the UPGMA method based on Jaccard’s distance coefficient. The accessions were classified into six groups based on the ISSR clustering pattern (Fig. 2a). The minimum genetic distance (0.16) was observed between G19 and G20 accessions, belonging to Qazvin Province, whereas a maximum genetic similarity of 0.78 was observed between G43 and G29, and also between G30 and G2 accessions.

**Fig. 3:**
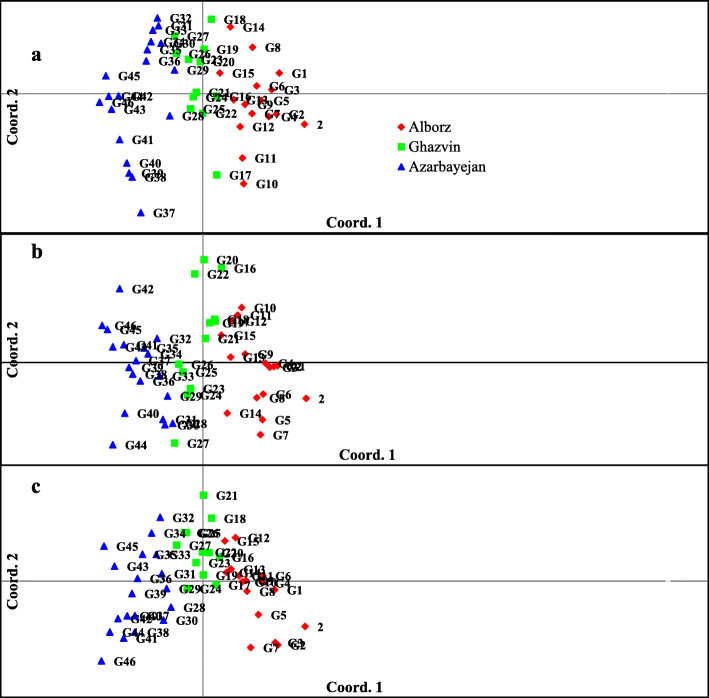
Bi-plots derived from the PCoA of 48 *Ae. triuncialis* accessions using ISSR (**a**), SCoT (**b**), and CBDP (**c**) data (1-40 are G1-G48 accessions)

The accessions were grouped into five clusters using SCoT data. The maximum genetic distance (0.75) was observed between G39 and G6, whereas the minimum (0.31) was observed between G36 and G35 accessions. Based on the SCoT marker, the accessions in each group showed a considerable amount of diversity, owing to their geographical distribution (Fig. 2b). The dendrogram generated by CBDP data showed the presence of five clusters (Fig. 2c). The genetic distances ranged from 0.03 (between G12 and G13) to 0.97 (between G16 and G47), with an average of 0.54. According to the results, most of the Alborz accessions were clustered into a group based on the dendrogram generated by each marker, including ISSR, SCoT, and CBDP. In addition, Qazvin accessions formed another group, although some of the accessions collected from this Province were categorized in other groups as well. Similar to the Alborz and Qazvin accessions, the accessions that were collected from East and West Azerbaijan Provinces were allocated to one group. These results were also observed when combining the data of the three markers (Fig. 2d). To determine the genetic relationships among and within the collected populations, PCoA was used. According to the results of the above analysis, the first two PCoA axes in ISSR, SCoT, and CBDP markers confirmed 77.28, 78.03, and 76.67% of the total variation among the studied accessions, respectively. The bi-plots confirmed the results of cluster analyses for the three markers (Fig. 3). In both analyses, the patterns of variation correspond to geographical distribution of the *Ae. triuncialis* accessions.

### Genetic variation revealed by the markers

According to ISSR data, the highest number of alleles (1.770) and Shannon index (0.393) were observed in accessions collected from the northwest of the country, whereas the lowest of these indices were in accessions collected from Qazvin Province. Also, the highest PPL (86.18%) belonged to northwestern populations (Table 5).

**Table 5:** Genetic variation features estimated using ISSR, SCoT, and CBDP markers in *Ae. triuncialis* populations

Parameters	ISSR			SCoT			CBDP		
	Northwest	Ghazvin	Alborz	Northwest	Ghazvin	Alborz	Northwest	Ghazvin	Alborz
PPL (%)	86.18	70.39	80.26	77.55	63.27	70.07	89.58	65.63	83.33
Na	1.77 ± 0.03	1.52 ± 0.03	1.65 ± 0.03	1.59 ± 0.04	1.37 ± 0.04	1.46 ± 0.04	1.66 ± 0.04	1.45 ± 0.04	1.79 ± 0.04
NA	1.43 ± 0.01	1.36 ± 0.01	1.44 ± 0.01	1.29 ± 0.02	1.29 ± 0.02	1.36 ± 0.02	1.55 ± 0.02	1.39 ± 0.02	1.51 ± 0.02
I	0.39 ± 0.01	0.32 ± 0.01	0.39 ± 0.01	0.29 ± 0.012	0.28 ± 0.012	0.33 ± 0.012	0.45 ± 0.01	0.35 ± 0.01	0.44 ± 0.01
He	0.25 ± 0.01	0.21 ± 0.01	0.25 ± 0.01	0.18 ± 0.01	0.18 ± 0.01	0.22 ± 0.01	0.31 ± 0.01	0.23 ± 0.01	0.26 ± 0.01

*PPL* percentage of polymorphic loci, *Na* observed no. of alleles, *Ne* effective no. of alleles, *I* Shannon index, *He* heterozygosity

Based on the SCoT markers, the highest (77.55%) and lowest (63.27%) PPL were attributed to accessions of the northwest and Qazvin Province, respectively. The highest and lowest values of Shannon information index were observed in populations of Alborz and Qazvin Provinces, respectively. For CBDP markers, the Na value varied from 1.45 to 1.79 (in Qazvin and northwest populations, respectively). The maximum (0.31) and minimum (0.23) Shannon index were observed in Alborz and Qazvin populations, respectively. The highest (89.58%) and lowest (65.63%) of PPL were found in the northwest and Qazvin populations, respectively. The average of PPL (79.51%) indicated a proper distribution and usefulness of the markers.

## Discussion

Studying genetic diversity among wild wheat species is important in breeding programs that involve interspecific crossings or targeted gene transfer in efforts to sustain and improve plant traits [33]. Landraces and wild crops can have unique traits and wide ranges of distribution in the plant flora in different geographical areas [34]. Therefore, assessments of genetic diversity among and within the wild relatives of bread wheat can be valuable steps before their evaluation in terms of resistance to biotic and abiotic stress. So far, few studies have been carried out on genetic diversity within populations of the *Aegilops* species, with a focus on valuable genes that express tolerance to different types of stress such as drought [35], heat [36], salinity [36], pests, and diseases [5]. In this study, three marker systems were used for investigating the genetic diversity among Iranian *Ae. trianiensis* accessions. To the best of our knowledge, this is the first report on genetic diversity among *Ae. triuncialis* accessions using two new markers, i.e., SCoT and CBDP, along with ISSR markers. In the present study, a high level of polymorphism was observed among the accessions in which the polymorphism percentages for SCoT, CBDP, and ISSR markers were 90.57, 94.74, and 93.97, respectively. The SCoT and CBDP markers were regarded as suitable for studying genetic diversity in wheat [37]. The ISSR primers can target microsatellites that are abundant throughout the plant genome. Thus, the markers have proven to be more reproducible than other markers such as RAPD [38]. However, the results of the present study showed that the CBDP markers, despite its ability to produce fewer bands, had the highest percentage of polymorphism in comparison with other markers (94.74%). The average PIC value for the CBDP primers was also higher than the other marker systems. Botstein et al. [39] stated that primers with a PIC value ranging from 0.25 to 0.50 contain useful information for genetic diversity studies. In agreement with our findings, Heikrujam et al. [14] reported that in terms of the PIC value, CBDP markers are more effective than SCoT markers when studying genetic diversity among male and female jojoba genotypes. Furthermore, other informative indices including Rp and MI showed a great approval of the discriminating power of these three markers. The higher values of MI and Rp in SCoT-24, SCoT-12, SCoT-9, and SCoT-4 primers indicated a better resolution and higher potency of these primers, as compared to other SCoT primers, which could be useful in future studies in *Aegilops* species. Etminan et al. [40] used ISSR and SCoT marker systems to detect genetic diversity in durum wheat genotypes. The authors indicated that Rp and MI could be the most important indices in determining marker efficiency, while the ISSR had a higher resolution than SCoT markers, as this was consistent with our findings. Also, the results of the present study confirmed the usefulness of these markers for identifying the genetic diversity of wheat and of its wild relative genotypes [41–43].

According to the Shannon index, the Alborz population had a higher variation than the other populations, which was confirmed by heterozygosity values.

All three marker systems showed the highest PPL in the northwestern genotypes, indicating a high allelic diversity and the heterogeneity of these genotypes. Our results confirmed that the main center for the diversity of the genus *Aegilops* in Iran was most probably located in the north and northwest of the country [44].

The dendrograms obtained from the three sets of data were roughly in line with the geographical origin of the samples. Reddy et al. [45] claimed that the accurate selection of ISSR primers could reveal an appropriate estimation of genetic variation to identify and classify data.

Baranduzi et al. [46] studied genetic diversity of *Aegilops* species using ISSR primers. Their samples were separated into six distinct clusters, which did not have any relationship with the geographical distribution pattern. In the present study, this relationship was based on ISSR and SCoT data and happened to be greater than CBDP data. The polymorphism in CBDP is due to the variation of the CAAT box in eukaryotic genomes [47]. Therefore, it seems that the accessions in distant geographic regions may have the same gene pool. Etminan et al. [48] reported that the genetic diversity among Iranian durum wheat germplasms was not in accordance with the geographical distribution, and that all accessions might be grouped based on their genomic structure. Fathi et al. [49] analyzed the genetic variation among *Ae. triunsialis* accessions by 5 IRAP markers. The authors observed a low level of relationship between genetic divergence and geographical origins of the samples. The three markers in the present study, i.e., ISSR, SCoT, and CBDP, were more appropriate for studying genetic diversity among and within *Aegilops* species, as compared to the IRAP markers. In the present study, different results and clustering were obtained by the three markers, due to the ability of each marker to reproduce different regions of the genome [16]. Therefore, these three markers provide more detail and diverse information about genetic diversity among and within the Iranian *Ae. triuncialis* accessions [50]. Mismatching results among the dendrograms being generated by different markers in different plants were indicated, such as in snake melon [51], sponge gourd [52], and bamboos [53]. The results of PCoA plots for the three marker systems confirmed the results of cluster analyses. The three bi-plots separated the samples according to their geographical locations. These results again demonstrated the ability of the three marker systems to identify the genetic diversity among the Iranian *Ae. triuncialis* accessions.

## Conclusion

The results of the present study revealed a high level of polymorphism in the Iranian *Ae. triuncialis* accessions by the three marker systems. Also, the results confirmed the efficiency of ISSR, SCoT, and CBDP markers in estimating the genetic diversity among the accessions. The three marker systems showed a comprehensive pattern of the genetic diversity among the Iranian *Ae. triuncialis* accessions, which could provide a future insight into wheat breeding programs.

## Data Availability

All data generated or analyzed during this study are included in this published article.

## Abbreviations

CBDP: CAAT box-derived polymorphism
CWRs: Crop wild relatives
H: Nei’s gene diversity
I: Shannon index
ISSR: Inter-simple sequence repeats
MI: Marker index
PCoA: Principal coordinate analysis
PIC: Polymorphic information content
PPL: Percentage of polymorphic loci
Rp: Resolving power
SCoT: Start codon targeted
UPGMA: Unweighted pair group method with arithmetic mean algorithm.
